# Progress in Surgery

**Published:** 1897-09-11

**Authors:** 


### PROGRESS IN SURGERY.

### ABDOMINAL SURGERY.

(*Continued from page* 393.)

**Intestinal Anastomosis.**—The pendulum of treatment still swings. Whilst some surgeons advocate mechanical media for connections, more particularly the inventors of the various buttons, &c., others still prefer simple suture and fall back upon the older method, which, when successful, is very successful, and does not give the surgeon subsequent anxiety. Two such cases are noted by Mr. John W. Taylor[17]: one, an excision of the cæcum for tuberculous disease in which the anastomosis was done by a continuous suture; the other, a posterior gastro-enterostomy for pyloric obstruction previously treated by Loreta's operation. Both operations took a long time to perform.

As a set off to the above may be mentioned a case of gastro-enterostomy with Murphy's button by Dr. Charles Briddon.[18] The patient lived 20 days, and post-mortem the button was found lying free in the stomach. The ilium had been united to the stomach by mistake, and it is said that on this account the patient on one occasion "vomited the nutrient enema almost unchanged."

Mr. Herbert Allingham performed enteroplasty on an apparently simple stricture of the small intestine about 2 ft. from the cæcum. Some months afterwards symptoms recurred, when the growth proved on operation to be malignant and irremovable. Intestinal anastomosis was accordingly performed with a bone bobbin with success.

New methods of intestinal anastomosis continue to be described. A rapid method has been devised by Dr.

Souligoux,[19] which consists in anastomosing the two portions of the digestive tube, without opening them, establishing a slough by crushing the convexity of each loop to be anastomosed over a limited and defined area, about four centimetres for each "contuse area." The loops are partially united outside these areas, then the contuse areas are cauterised with caustic potash, and the suturing is then continued around. It takes about 48 hours for the slough to separate and form a channel, and in that time the peritoneal adhesions are usually safe. Dr. Chaput has modified this operation by discarding the crushing, and using the actual cautery.

Dr. J. Frank,[20] of Chicago, has devised a form of button, which, however, he has not used on a human patient yet. It is a combination of the principle of Murphy's button with the bone bobbin. Two decalcified bone collars are sewn on to a piece of india-rubber tubing so that they meet in the centre and can be separated by pulling upon them, thus stretching the india-rubber tubing, which corresponds in principle with the spring of a Murphy's button. Later on the collars dissolve, and the elastic tubing is set free into the intestine.

**Fæcal Fistula.**—Dr. Kammerer[21] has given details of the method of treatment in these cases by completely detaching and excluding the part of the bowel connected with the fistula, and performing a short circuit anastomosis of the intestines. The operation seems particularly applicable to the cure of fæcal fistulæ connected with the cæcal region. Partial exclusion by anastomosis alone may have to be supplemented by complete exclusion to ensure a cure. Murphy's button was used in the cases quoted.

Dr. George Wm. Perkins (Utah)[22] describes a case of fæcal fistula of the cæcum which he cured by excising the fistula and sewing up the gut.

Dr. John Rushmore (Brooklyn)[23] attributes many cases of fæcal fistula in the cæcal region to careless, bad suturing, and the use or too prolonged use of rubber drainage tubes, causing ulceration into the bowel.

**Meckel's Diverticulum.**—A rare condition, probably unique, has been published by Dr. Beach (Boston).[24] The patient passed fæcal material, with starch granules, with the urine. At the operation a tumour was found between the bladder and ilium, opening up into each, and thought to be a calcified Meckel's diverticulum containing a fæcal concretion. In some respects the case reads like a dermoid of the urachus communicating with the ilium, but in favour of it being a Meckel's diverticulum is the fact that the cyst wall had the microscopic glandular aspect of small intestine.

[17] Birmingham Med. Review, Feb 1897. [18] Annals of Surgery, Jan., 1897. [19] La Presse Médicale, 1896, No. 59. [20] Med. Record, Oct. 3, 1896. [21] Med. Record, Feb. 20, 1897. [22] Annals of Surgery, Dec., 1896. [23] Annals of Surgery, Oct., 1896. [24] Annals of Surgery, Oct., 1896.

---

### GENERAL SURGERY.

(*Continued from page 394.*)

**Fractures and Dislocations.**—Fracture of the malar bone is so infrequent, its depression into the antrum yet more so, and the deformity so horrible, if nothing is done to replace the broken bone, that the operation recently adopted by R. F. Weir[14] is worthy of consideration. The measure adopted in two instances consisted in making a small opening with a gouge into the antrum within the mouth, just above the canine teeth, so that through this a No. 12 or 14 (English) sound could be introduced, and by this means the depressed fragment forced upwards and outwards into its proper position. A plug of iodoform gauze was inserted in the antrum to retain the connected position; this was withdrawn on the tenth day. The result in each case was very satisfactory, all deformity being absent when the patients were seen several months later. Dr. Abbe[15] had a similar case of fracture of the malar bone. It occurred in a young man whose head had come in collision with that of another man while playing at football. After elevating the depressed fragment with a probe introduced under the lip, Abbe supported it with a drill passed through the solid part of the zygoma. The drill was removed on the fourteenth day, and the bone found to be solidly united. By this simple procedure the antrum was preserved from possible infection, although it had the disadvantage of leaving a small puncture scar.

**Upper Extremity.**—Whatever may be the site of fracture of the clavicle, and whatever may be the cause, experience has taught us that the ordinary treatment by bandages and slings is, in many cases, unsatisfactory, on account of the difficulty of keeping the fragments fixed and in good position. Considerable callus is usually present and often deformity, while the functions of the shoulder and elbow are frequently impaired from the prolonged fixation. The recumbent position, with a pad between the shoulders and a small sand-bag over the prominent fragment, acts well in many cases; but when the fragments are many, and when pain is persistent, G. W. Spencer[16] recommends treatment by incision and suture. He records two cases in which this was carried out, and advocates the employment of a ligature of kangaroo tendon instead of silver wire to hold the fragments in apposition. The result in both cases was most satisfactory. M. M. Franklin[17] publishes a case of double fracture of the clavicle from the kick of a horse's hoof. The inner fracture was transverse, about one inch from sternum, within the fibres of the rhomboid ligament, causing very little deformity; the other was a very oblique fracture, beginning at the lower border of the outer third, and ending at the upper border of the acromial end. The sharp outer end of the middle fragment had pierced all the tissues except the skin, which being badly contused required little to cause the fracture to become compound. This occurred the next day. Wiring was deemed inadvisable, because of the contusion of the tissues, which threatened to slough; because of the possibility of the union of the fractures, and the closure of the wound by granulation. Velpeau's bandage was applied, and at the end of four weeks there was complete union of the inner with but partial of the outer. The outer end of the middle fragment, which projected through the skin, was now resected, and the arm still retained for three weeks in the bandage. The patient was then allowed to use the arm carefully, both fractures by this time having united solidly. Dr. Brewer[18] records the case of a man with most marked deformity following a fracture of the clavicle. A large amount of callus had formed at the angular junction of the fragments, and had involved the brachial plexus, producing partial paralysis of the arm. He removed the callus and brought the divided ends of the clavicle—separated by at least an inch — together with wire. The paralysis disappeared, and the result was in every way successful, although the clavicle was shortened more than an inch. Dragon[19] gives the results of massage applied to 20 cases of fracture of the clavicle under the care of Lucas-Championnière. The author practises a daily massage, not only at the seat of fracture, but also of the adjacent joints and muscles, and insists particularly on massage of the deltoid muscles, and careful movements, both active and passive, of the shoulder. In the intervals the upper limb is supported in a sling. At the New York Academy of Medicine, Dr. Fisk[20] presented an aged man, with a somewhat rare dislocation of the outer end of the clavicle very far backwards. It had occurred eight months previously, and great difficulty was experienced in keeping the bone reduced. A rare instance of simultaneous dislocation of both ends of the clavicle with fracture of the scapula is recorded by Dr. W. F. Gibb.[21] It was in a man aged 64, who had fallen four and a half feet on to his left shoulder. Death took place on the eighth day from bronchitis. M. C. Walther[22] relates in detail two cases of successful operation for traumatic displacement of the upper epiphysis of the humerus. In the first case, which was one of recent injury, after an attempt to reduce the displacement by extension of the upper limb, the seat of injury was cut down, and the upper end of the diaphysis resected. In the second, the displaced epiphysis had been fixed to the diaphysis by a large mass of callus. The upper end of the shaft was divided and resected, and the projecting portion of callus removed. The results in both cases were satisfactory, both as regards the removal of the deformity and the restoration of the functions of the arm. Dr. J. B. Roberts[23] gives as his opinion that non-union is more common in fractures of the shaft of the humerus than in other bones, because of the entanglement of the soft tissues between the fragments. He mentions a case of fracture at the junction of the upper and middle third of the humerus, in which it was impossible to reduce the fragments even after exposing the bone by an incision. The stripped-up periosteum and the muscles held such relations to the fragments that the continuity of the bone could not be restored until resection of the ends had been performed. This surgeon, in 1892, advocated (" Trans. Amer. Surg. Assoc.," Vol. X.) exploratory incision in a large number of recent fractures of the lower end of the humerus, where satisfactory coaptation was not attainable under anæsthesia. In a more recent paper,[24] he sets forth a further plea for the wider application of an open operation in cases of simple fracture and dislocation. If this is to be adopted, it is evident that the patient will receive the greatest advantage if it be undertaken before the head of the bone or its socket be altered in shape, and muscles and fasciæ become contracted and adherent to surrounding tissues. Any structures resisting reduction can be divided. Ankylosis is more liable to occur from displaced fragments or articular surfaces, irregular callus formation, and interference with articular contact than from aseptic incision and readjustment of the joint structures. Professor Kocher[25] has lately published an excellent book on the principal fractures of the humerus and femur. The object of this work is to afford the practitioner an exact clinical description f the most important and frequent fractures of these bones, whilst others of minor importance are not neglected. The first part treats of fractures of the upper end of the humerus as supratubercular, *e.g.*, fracture of the anatomical neck, and infratubercular. The latter group he divides into pertubercular, *i.e.*, epiphysial fractures, or fractures about the epiphysial line, and subtubercular fractures, viz., those which occur a little lower down. The second part comprises fractures of the lower end of the humerus—divided into the following seven varieties: (1) supracondylar fractures; (2) fractures of the external condyle; (3) fracture of the internal epicondyle (epitrochlea); (4) diacondylar fracture (*i.e.*, transverse fracture across the epicondyle and the epitrochlea); (5) fracture of the internal condyle (trochlea); (6) fracture of the external epicondyle; (7) partial fracture of the articular surface of the condyle. In addition to these, there are also the complex fractures known as T-shaped and Y-shaped fractures. The third part deals with fractures of the upper end of the femur. Kocher here makes two groups—supratrochanteric and the infratrochanteric. The first are divided into (1) fractura subcapitalis, *i.e.*, the intra capsular fractures of previous nomenclature; and (2) the intertrochanteric, *i.e.*, those which are placed nearer the intertrochanteric lines (mostly extra capsular). The second group he divides into pertrochanteric fractures, *i.e*, through the region of the two trochanters; and infratrochanteric, *i.e.*, below the trochanters. Some fractures of the lower end of the radius, as seen by the Röntgen rays, are described by Leo Freeman.[26] One was the case of a boy aged ten years, diagnosed from the "silver fork" deformity as an epiphysial separation. The skiagraphs taken 14 weeks after the accident made it at once clear why no amount of force or method of manipulation would reduce the deformity. In the prone position of the forearms the epiphysial line of each radius was seen to be the same width throughout, one bone being apparently as perfect as the other. In the semiprone position, however, the anterior half of the right epiphysial line was obscured by a dark shadow, representing, undoubtedly, a growth of new bone, and the anterior border of the epiphysis was tilted downwards from the shaft. Freeman thought that the abnormal position of the bones was due to stimulated growth and not to inhibited growth, although the latter might take place later. In a second case, in a lady aged 52 years, there was revealed a rare form of fracture of the lower end of the radius, viz., a splitting off of the styloid process, together with a portion of the shaft. It had been diagnosed as a Colles's fracture. In a third case, in a boy aged six, both bones of the forearm were broken three-quarters of an inch above the epiphysial lines. The skiagraph showed that the fragments were far from being accurately adjusted. Resetting of the fragments was suggested, but refused; and, in spite of its being left, the wrist became almost perfect both as regards appearance and function.

[14] N.Y. Medical Record, March 6, 1897, p. 335. [15] Ibid., p. 348. [16] Amer. Jour. of Med. Sciences, April, 1897, p. 445. [17] Annals of Surgery, June, 1897, p. 716. [18] N.Y. Medical Record, Feb. 20, 1897, p. 280. [19] Jour. de Méd. et de Chir. Prat.; and Epit. Brit. Med. Jour., March 20, 1897, p. 46. [20] N. Y. Medical Record, March 20, 1897, p. 425. [21] Lancet, Feb. 6, 1897. [22] Revue d'Orthopédie, No. 1, Jan., 1897, p. 39. [23] Annals of Surgery, May, 1897, p. 638. [24] Medical Chronicle, Feb., 1897; and N. Y. Med. News, Jan. 16, 1897. [25] Beiträge zur Kenntniss einiger praktisch wichtiger Fracturformen. [26] Annals of Surgery, April, 1897, p. 470.

